# Mathematical Models for Ultrasound Elastography: Recent Advances to Improve Accuracy and Clinical Utility

**DOI:** 10.3390/bioengineering11100991

**Published:** 2024-09-30

**Authors:** Ali Farajpour, Wendy V. Ingman

**Affiliations:** 1 Adelaide Medical School, University of Adelaide, The Queen Elizabeth Hospital, Woodville South, Adelaide, SA 5011, Australia; ali.farajpourouderji@adelaide.edu.au; 2 Robinson Research Institute, University of Adelaide, Adelaide, SA 5006, Australia

**Keywords:** ultrasound elastography, continuum models, mechanical properties, biomedical imaging, disease diagnosis

## Abstract

Changes in biomechanical properties such as elasticity modulus, viscosity, and poroelastic features are linked to the health status of biological tissues. Ultrasound elastography is a non-invasive imaging tool that quantitatively maps these biomechanical characteristics for diagnostic and treatment monitoring purposes. Mathematical models are essential in ultrasound elastography as they convert the raw data obtained from tissue displacement caused by ultrasound waves into the images observed by clinicians. This article reviews the available mathematical frameworks of continuum mechanics for extracting the biomechanical characteristics of biological tissues in ultrasound elastography. Continuum-mechanics-based approaches such as classical viscoelasticity, elasticity, and poroelasticity models, as well as nonlocal continuum-based models, are described. The accuracy of ultrasound elastography can be increased with the recent advancements in continuum modelling techniques including hyperelasticity, biphasic theory, nonlocal viscoelasticity, inversion-based elasticity, and incorporating scale effects. However, the time taken to convert the data into clinical images increases with more complex models, and this is a major challenge for expanding the clinical utility of ultrasound elastography. As we strive to provide the most accurate imaging for patients, further research is needed to refine mathematical models for incorporation into the clinical workflow.

## 1. Introduction

Medical imaging has a critical role in disease detection and diagnosis and helps to direct and monitor treatment efficacy [1,2,3,4]. The development of improved imaging technologies that can detect diseases such as breast cancer, liver fibrosis, or vascular diseases at an early stage has the potential to provide a wider range of therapy options with less undesirable side effects [5,6,7]. This can lead to enhanced treatment efficacies, less invasive procedures, and faster recovery periods. Accuracy is a key aspect of imaging; false positives can lead to unnecessary interventions such as biopsies or surgeries. On the other hand, false negatives can result in delayed therapy with a poorer prognosis [8,9]. From an economic point of view, early disease detection is an important factor in lifting the financial burden off patients’ shoulders and reducing the overall healthcare system costs at the national level. Developing accurate imaging technologies and providing access to them through public health screening reduces healthcare costs while improving patient outcomes [10,11].

Mechanical properties of biological tissues such as elasticity modulus and fluid viscosity can experience significant alterations when disease is present [12,13]. The elasticity modulus is defined as the ratio of mechanical stress per strain in the uniaxial compression test and is an indicator of tissue stiffness. When external mechanical loading is exerted on biological tissue, the elasticity modulus together with other factors such as Poisson’s ratio and fluid viscosity dictates how the tissue deforms. Overall, it has been indicated that the elasticity modulus of solid tumours is significantly higher than that of healthy tissue at large-scale levels [14,15]. Moreover, mechanical stiffness has been used to detect liver and ovarian diseases as tissue fibrosis is highly associated with elasticity modulus [16]. Even in diffuse diseases such as chronic hepatitis, mechanical parameters reflect changes in fibrous patterns induced by hepatitis progression, indicating the stage of the disease [17]. In addition, the alteration of elastic and viscoelastic properties of arterial walls subject to atherosclerosis provides useful information regarding plaque composition and the risk of cardiovascular diseases [18]. Recent studies show that Poisson’s ratio, which is defined as the transverse-to-axial strain ratio, not only provides insight into disease status and stage but also improves the accuracy of the estimation of elasticity modulus within biological tissue [19].

Elastography is a biomedical imaging tool that enables the estimation of mechanical properties of biological tissues and the visualisation of them on images known as elastograms [20]. This technique provides a non-invasive, relatively rapid, and easy-to-use method to quantitatively assess tissue health status. To date, elastography techniques have been used in assessing a wide range of diseases including, but not limited to, breast cancer [21], liver fibrosis [22], prostate cancer [23], and vascular [24] and muscular [25] disorders. Elastography imaging is classified according to the approach used to induce mechanical deformation or/and the method of measuring displacement distribution. When ultrasound waves are generated and propagated within the biological tissue of interest, it is called ultrasound elastography [26]. When magnetic resonance imaging is combined with a vibrating device to generate deformation, elastograms over a broader organ region are obtained and magnetic resonance elastography is achieved [27]. Optical coherence elastography is another common type of elastography imaging utilised in the clinic, where displacement measurements often rely on optical coherence tomography [28]. This technique provides relatively higher resolution at a specific localised region of biological tissue. However, it is restricted in terms of the penetration depth and best used in assessing the mechanics of superficial tissue where in-depth visualisation is not required [29].

Elastography for biomedical imaging applications is a fast-growing area of research, with a number of review papers published in recent years. Wu et al. [30] conducted a systematic review and meta-analysis in order to study the application of ultrasound elastography for the assessment of plantar fasciitis. They found that patients with plantar fasciitis have a less shear wave speed than those with no plantar fascia inflammation. Caenen et al. [31] reviewed and studied the application of ultrasound shear wave elastography in the detection of cardiovascular disorders. They concluded that ultrasound elastography has shown great promise as an imaging tool in cardiology; however, accurate interpretation and use of shear wave elastography images require further development of mechanical equations and advanced metrics. Additionally, a number of valuable review papers have been recently published on the application of ultrasound elastography in the assessment of adult skeletal muscle [32], neurosurgery [33], chronic kidney disease [34], uterus and endometrium disorders [35], head and neck cancers [36], breast cancer [37,38], evaluation of lymph nodes [39], brain diseases [40], and inflammatory bowel diseases [41]. To the best of our knowledge, no review paper has been published on the development of continuum mechanics models for ultrasound elastography imaging.

In all of the above-mentioned elastography technologies, it is essential to extract the intrinsic mechanical properties such as elasticity modulus, viscoelastic constant, and Poisson’s ratio from displacement and force measurements using accurate mathematical frameworks. The resolution and sensitivity of elastography imaging depend not only on measurement preciseness but also on the accuracy of the mathematical models used to estimate the mechanical characteristics of the tissue. Different continuum models including elasticity, viscoelasticity, poroelasticity, and nonlocal models have been developed since the first emergence of elastography in the 1990s [42,43] to estimate mechanical properties and produce quality elastography images. In this review paper, different continuum modelling approaches used in ultrasound elastography imaging are presented and each model’s benefits and drawbacks are summarised. Particular attention is made to the most recent advances in continuum modelling strategies, highlighting current research gaps and essential requirements to improve the accuracy of mathematical modelling. This review would be beneficial for biomedical engineers, biologists, and clinical practitioners who aim to develop and use elastography devices for medical imaging.

## 2. Fundamental Concepts

Classical elasticity theory is formulated based on the assumption that mechanical stress at a given point is a function of the strain components at that point only. In this theory, mechanobiological parameters such as the viscosity effect of the fluid content of the tissue and fluid–solid interactions within the tissue are not taken into account. In a one-dimensional domain, the strain–stress relation of the classical elasticity theory (neglecting the material and geometrical nonlinearities) is given by

(1) $$\sigma_{xx}^{cl}=E\varepsilon_{xx},$$

where $\sigma_{xx}^{cl}$ and $\varepsilon_{xx}$ are, respectively, the mechanical stress and strain along the x axis (the loading direction). *E* is the elasticity modulus, which is an indicator of the tissue stiffness in a specific region. In sections with higher stiffness, the elasticity modulus is higher, indicating that the tissue exhibits a small degree of deformation under mechanical stress. By contrast, regions with lower stiffness have lower elasticity moduli and thus display larger deformation when external mechanical loading is applied. This simple equation (Equation (1)) forms the basic foundation of elastography imaging. In many diseases such as liver fibrosis, breast cancer, and muscular disorders, elasticity modulus is changed as a consequence of the disease progression. As a rule of thumb, particularly in solid tumours and fibrosis-based diseases, the elasticity modulus is significantly higher in abnormal lesions compared to healthy tissue at large scales [15,22]. In this context, large scales denote the tissue size, which is in the order of several millimeters to centimeters while any size less than one millimeter is referred to as ‘small-scale level’.

In Equation (1), the axial strain component is defined as $\varepsilon_{xx}=\left(l-l_{0}\right)/l_{0}$ where $l_{0}$ is the initial length of the one-dimensional tissue before deformation whereas l represents tissue length after deformation. In a sub-class of elastography imaging known as strain elastography, strain components like $\varepsilon_{xx}$ are visualised to detect regions with less deformation (rigid regions), which might have some clinical information on disease status. Although the strain parameter is an important feature in the biomechanical analysis of tissue deformation, it is not recommended to be used as the main parameter in elastography for clinical purposes. This is because strain is not an intrinsic biomechanical property, and thus it is not an indicator of the actual stiffness of the tissue. Indeed, it describes the geometrical deformation of the tissue under mechanical loading by mathematically comparing the size of the biological tissue before and after the loading process.

The axial mechanical stress is defined as the ratio of axial force per unit area and can be mathematically expressed by *F*/*A* where *F* and *A* are the axial force and cross-sectional area of the biological tissue, respectively. One of the big challenges in almost all elastography imaging techniques is to accurately estimate the distribution of stress components within the tissue. We only have information about the stress on the surface of the tissue and not the spatial distribution of the stress inside the target tissue. This distribution generally depends on a number of different factors such as tissue geometrical shape, boundary conditions, and external loading. In order to calculate the intrinsic elasticity modulus of biological tissue under external mechanical loading, an accurate estimation of the stress distribution is key. In elastography, the calculation of strain components is usually performed by the analysis of a sequence of images of the tissue structure before and after deformation. Then, all generated images are registered and aligned to allow for precise tracking of each point within the tissue. Stress distributions, on the other hand, enable us to use constitutive equations including Equation (1) to obtain the intrinsic elasticity modulus.

**Figure 1:**
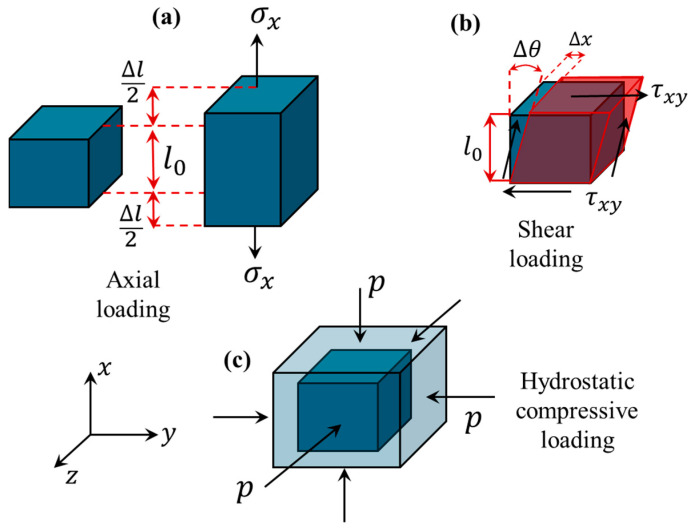
Three different loading conditions used in ultrasound elastography imaging: (**a**) axial loading, which can be conducted using either compressive load or tensile load, (**b**) shear loading, and (**c**) hydrostatic compressive loading.

In addition to axial loading (Figure 1a), fundamental elements of biological tissue can be exposed to shear loading (Figure 1b) and hydrostatic compressive loading (Figure 1c). In the shear loading condition, a shear force of magnitude *F*_s_ is applied on the top surface of the biological tissue element as shown in Figure 1b. The shear stress is obtained by dividing the shear force over the top surface area A as $\tau_{xy}^{cl}=F_{s}/A$. On the other hand, according to Hook’s law for shear stress, we have $\tau_{xy}^{cl}=G\gamma_{xy}$ where G and $\gamma_{xy}$ are the shear modulus and engineering shear strain, respectively. $\gamma_{xy}$ is defined by $\gamma_{xy}=\Delta x/l_{0}$ in which $l_{0}$ and Δx are the initial length of the cubic element and the displacement induced by the shear loading (refer to Figure 1b). For small deformation, we have $\gamma_{xy}=tan(\Delta \theta )\approx \Delta \theta$ where Δθ denotes the angular deformation. It should be noted that the engineering shear strain is different from the corresponding shear strain tensor. The shear component of the strain tensor is related to the engineering shear strain as $\varepsilon_{xy}=\gamma_{xy}/2$. Shear components in the strain tensor are half of their corresponding engineering shear strains. Another important elasticity constant is the bulk modulus, which is associated with hydrostatic compressive loading (Figure 1c). In this loading condition, the biological cubic element is subject to equal pressure along all three directions, causing volumetric reduction (volume strain, $\varepsilon_{v}$). The hydrostatic pressure (p) is proportionally related to the volume strain by $p=K\varepsilon_{v}$ where K is the bulk modulus. The volume strain represents the change in the volume of the element and is defined by $\varepsilon_{v}=\Delta V/V$. The bulk modulus and elasticity modulus are not independent mechanical variables. They are related by the following equation, K=E/[3(1−2v)], in which v is Poisson’s ratio. It is worth mentioning that the shear modulus also relates to the elasticity modulus by G=E/[2(1+v)]. Table 1 lists the three different mechanical loading conditions with their relevant properties and formulas.

**Figure 2:**
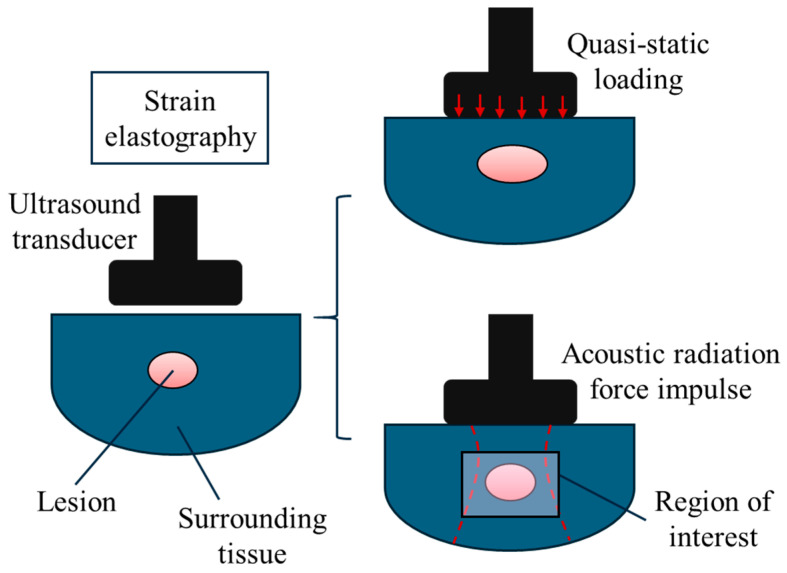
Strain ultrasound elastography imaging can be conducted through two common approaches: (1) quasi-static loading, and (2) the acoustic radiation force impulse.

**Figure 3:**
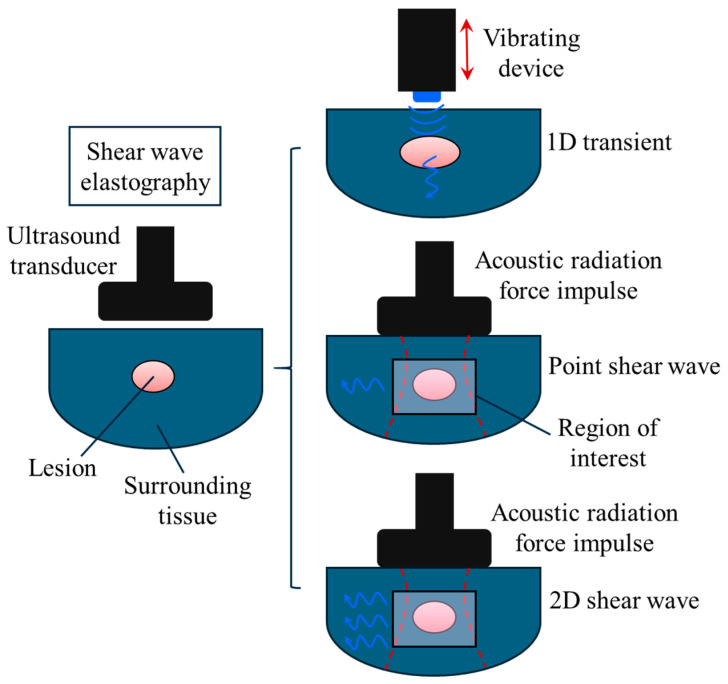
Three techniques of shear wave ultrasound elastography: (1) one-dimensional (1-) transient ultrasound elastography, (2) point shear wave ultrasound elastography, and (3) two-dimensional (2D) shear wave ultrasound elastography.

In general, ultrasound elastography can be divided into two main categories: (1) strain elastography and (2) shear wave elastography [21,26]. This classification is based on the method through which the biomechanical properties are extracted and visualised on an elastogram. For strain elastography, normal mechanical stress is applied to the tissue or organ and the consequent displacements within the region of interest are measured (Figure 2). In shear wave elastography, the speed of shear waves is measured and used for visualisation and analysis. To induce shear waves, dynamic stress is produced using mechanical vibrating tools, acoustic radiation force impulses, or a combination of the two as shown in Figure 3.

Strain elasticity imaging is classified based on the mechanism of the mechanical excitation. The biological tissue or organ can be stimulated using manually applied force on the top surface as shown in Figure 2, which is known as quasi-static strain elastography [26,45]. As an alternative method, short-duration acoustic pulses can be created in a controlled manner to induce mechanical loading within the tissue, which is known as acoustic radiation force impulse strain elastography. The displacement components are then measured using a variety of different techniques including radio frequency echo analysis, doppler, and cross-correlation speckle tracking. Mathematical models are used on this data to characterise the mechanical properties of the region of interest. The simplest mathematical model is to use Equation (1) and assume that the longitudinal stress is uniform within the tissue.

Shear wave ultrasound elastography can be classified according to the technique utilised to generate shear waves. When an external vibrating tool is applied, shear waves are created along the excitation direction as shown in Figure 3. This imaging technique is widely known as one-dimensional transient ultrasound elastography. By contrast, shear waves are induced perpendicular to the excitation axis in the acoustic radiation force method. The acoustic radiation force impulse-based shear wave elastography is divided into two main categories: (1) point shear wave and (2) two-dimensional shear wave [20,21,22,25]. In point shear wave ultrasound elastography, focused ultrasound pulses are generated in a particular point of interest within the biological tissue. However, multiple shear waves across the region of interest are generated in two-dimensional shear wave ultrasound elastography. This provides more details and a comprehensive overview of a broader area of the biological tissue. The basic Hooke’s law of all shear wave ultrasound elastography is $\tau_{xy}^{cl}=G\gamma_{xy}$; refer to Table 1 for more details about this equation.

**Figure 4:**
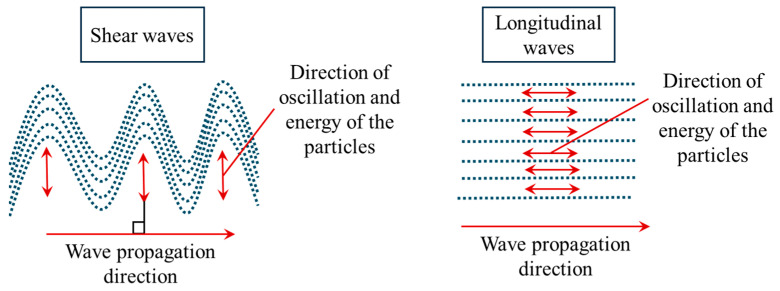
Two types of wave propagation systems are commonly used in ultrasound elastography: (1) shear waves, in which the direction of oscillation of particles of the biological tissue is perpendicular to the wave propagation direction, and (2) longitudinal waves where the direction of particles’ oscillation is parallel to the wave propagation direction. The former is used in shear wave elastography imaging while the latter is used in B-mode ultrasound and 1D transient elastography [20,47,48].

In addition to ultrasound elastography classification, mechanical waves can be also classified and grouped based on their mechanism of propagation and induced oscillation [46,47]. Two types of mechanical waves are widely used in ultrasound elasticity imaging: (1) shear waves and (2) longitudinal waves. When shear waves are generated, the direction of oscillation of particles is perpendicular to the propagation direction (Figure 4). By contrast, in longitudinal ultrasound waves, the propagation direction is parallel to the particle oscillation direction. In B-mode ultrasound and 1-dimensional transient ultrasound elastography, longitudinal waves are commonly utilised to extract the mechanical properties of the biological tissue whereas shear waves are generated and used to produce elastography images in shear wave ultrasound elastography. In Table 2, more details are provided regarding the longitudinal and shear waves used in elastography techniques.

The speed of longitudinal waves propagated in an elastic medium is in a direct proportional relationship with the square root of the bulk modulus. However, the longitudinal wave speed is inversely related to the mass density of the elastic body. The speed of shear waves in an elastic body is proportionally related to the root square of the shear modulus, as shown in Table 2. These mathematical expressions allow for the smooth and fast conversion between the wave speed and tissue stiffness. The approximate mean speed of longitudinal waves in soft tissue is around 1540 m/s while the shear wave speed in soft tissue is between 1 and 10 m/s [20]. The longitudinal wave speed is much higher than the shear wave speed and it is not highly sensitive to changes in the tissue elasticity constant. Shear waves are more desirable for elastography imaging as they are directly linked to the tissue shear modulus and more sensitive to stiffness alterations that are associated with disease. Indeed, slower propagation of shear waves compared to longitudinal waves allows for a more accurate and sensitive elasticity imaging assessment within biological tissue.

Moreover, the longitudinal wave mode is not compatible with the incompressible medium assumption. When the elastic medium is assumed to be incompressible, the Poisson’s ratio is about 0.5, which consequently results in an infinite bulk modulus. This hinders wave speed-elasticity conversion. By contrast, the previously proposed mathematical equation for conversion between shear elasticity and propagation speed of shear waves is still viable for incompressible materials. The shear modulus of incompressible tissues is one-third of their elasticity constant.

## 3. Classical Elasticity Theory

In a general form where there is no limitation on the dimensionality of the problem, the constitutive equation of the classical elasticity theory can be written by [44]

(2) $$\sigma_{ij}=C_{ijkl}\varepsilon_{kl}.$$

where $\sigma_{ij}$ is the component of the mechanical stress tensor that is along the *j* direction, acting on the plane perpendicular to the *i* axis. $\varepsilon_{kl}$ is the general strain component and $C_{ijkl}$ denotes the components of the elasticity tensor, containing all elasticity and shear moduli. According to Equation (2), the local mechanical stresses in a two-dimensional domain (*x*-*y* plane) are obtained as

(3) $$\sigma_{xx}=\left(\frac{E_{11}}{1-\nu_{12}\nu_{21}}\right)\varepsilon_{xx}+\left(\frac{\nu_{12}E_{22}}{1-\nu_{12}\nu_{21}}\right)\varepsilon_{yy},\;\sigma_{yy}=\left(\frac{\nu_{12}E_{22}}{1-\nu_{12}\nu_{21}}\right)\varepsilon_{xx}+\left(\frac{E_{22}}{1-\nu_{12}\nu_{21}}\right)\varepsilon_{yy},\;\sigma_{xy}=2G_{12}\varepsilon_{xy},$$

Here, $\nu_{ij}$ represents the tissue Poisson’s ratio, which is calculated by dividing lateral strain to the main strain along the direction of the stress ($\nu_{ij}=-\varepsilon_{jj}/\varepsilon_{ii}$). It is an indicator of how tissue deforms along the lateral direction with respect to its deformation along the main direction of the stress. Greater values of Poisson’s ratio are associated with higher lateral displacements. A recent study highlights the importance of the role of Poisson’s ratio not only in enhancing the visualisation of elastography images by providing extra clinical information on lateral deflection but also in improving the accuracy of the estimation of elasticity modulus within the tissue [19]. In Equation (3), $G_{12}$ is the shear modulus that is an intrinsic biomechanical property, indicating tissue’s resistance to shear forces. Shear wave elastography techniques have been developed to estimate this property within different tissue regions to detect any abnormalities associated with diseased conditions [49,50].

Assuming small deflection caused only by in-plane mechanical loading, the strain components in a two-dimensional environment can be expressed as

(4) $$\varepsilon_{xx}(x,y,z,t)=\frac{\partial u(x,y,t)}{\partial x},\;\varepsilon_{yy}(x,y,z,t)=\frac{\partial v(x,y,t)}{\partial y},\;\varepsilon_{xy}(x,y,z,t)=0.5\left(\frac{\partial v(x,y,t)}{\partial x}+\frac{\partial u(x,y,t)}{\partial y}\right),$$

where *u* and *v* are the displacements along the *x* and *y* directions, respectively. Now, stress resultants (*T_ij_*) can be calculated through integrating over the *z* direction

(5) $$\langle T_{xx},\;{\;T}_{yy},{\;T}_{xy}\rangle =\int_{-h/2}^{h/2}\langle \sigma_{xx},{\;\sigma }_{yy},{\;\sigma }_{xy}\rangle dz.$$

In the above relations, *h* is the tissue thickness. By substituting Equation (4) into Equation (3) and then inserting the resultant expressions for the in-plane stress components into Equation (5), we obtain

(6) $$T_{xx}=\left(A_{11}\frac{\partial u}{\partial x}+A_{12}\frac{\partial v}{\partial y}\right),\;T_{xy}=\left(A_{21}\frac{\partial u}{\partial x}+A_{22}\frac{\partial v}{\partial y}\right),\;T_{xy}=A_{33}\left(\frac{\partial u}{\partial y}+\frac{\partial v}{\partial x}\right),$$

where *A_ij_* indicates the tissue in-plane stiffness. As mentioned earlier, the elasticity modulus is an indicator of stiffness of the biological tissue. This association for the in-plane deformation can be mathematically described by

(7) $$\langle A_{11},A_{22},A_{12},A_{21}\rangle =\frac{h}{1-\nu_{21}\nu_{12}}\langle E_{11},E_{22},\nu_{12}E_{22},\nu_{12}E_{22}\rangle ,A_{33}=G_{12}h,$$

It is found that the elasticity modulus is in a direct proportional relation with the in-plane tissue stiffness. According to Hamilton’s principle, in-plane governing equations are

(8) $$\frac{\partial T_{xy}}{\partial y}+\frac{\partial T_{xx}}{\partial x}-m_{0}\frac{\partial^{2}u}{\partial t^{2}}=0,\;\frac{\partial T_{xy}}{\partial x}+\frac{\partial T_{yy}}{\partial y}-m_{0}\frac{\partial^{2}v}{\partial t^{2}}=0,$$

where $m_{0}=\rho_{bt}h$ in which $\rho_{bt}$ denotes the mass per unit volume of the biological tissue. Substituting Equation (6) into Equations (8) and (9) leads to the final equations of in-plane motion developed within the framework of the classical elasticity theory.

(9) $$A_{11}\frac{\partial^{2}u}{\partial x^{2}}+A_{12}\frac{\partial^{2}v}{\partial x\partial y}+A_{33}\left(\frac{\partial^{2}u}{\partial y^{2}}+\frac{\partial^{2}v}{\partial x\partial y}\right)=m_{0}\frac{\partial^{2}u}{\partial t^{2}},$$

(10) $$A_{33}\left(\frac{\partial^{2}u}{\partial x\partial y}+\frac{\partial^{2}v}{\partial x^{2}}\right)+A_{21}\frac{\partial^{2}u}{\partial y\partial x}+A_{22}\frac{\partial^{2}v}{\partial y^{2}}=m_{0}\frac{\partial^{2}v}{\partial t^{2}}.$$

Similarly, the governing equation of the biological tissue under the transverse deflection assumption can be derived using the classical elasticity. Assuming *w* is the transverse displacement and in-plane displacements are negligible, the strain components are

(11) $$\varepsilon_{xx}(z,y,x,t)=-\frac{\partial^{2}w(y,x,t)}{\partial x^{2}}z,\;\varepsilon_{yy}(z,y,x,t)=-\frac{\partial^{2}w(y,x,t)}{\partial y^{2}}z,\;\varepsilon_{yx}(z,y,x,t)=-\frac{\partial^{2}w(y,x,t)}{\partial y\partial x}z,$$

The couple resultants ($M_{ij}$) acting within the tissue, induced by the stress components, are defined by

(12) $$M_{ij}=\int_{-h/2}^{h/2}z\sigma_{ij}dz,$$

Using Equation (3) together with Equation (12), one can obtain the couple resultants

(13) $$M_{xx}=-D_{12}\frac{\partial^{2}w}{\partial y^{2}}-D_{11}\frac{\partial^{2}w}{\partial x^{2}},\;M_{yy}=-D_{22}\frac{\partial^{2}w}{\partial y^{2}}-D_{12}\frac{\partial^{2}w}{\partial x^{2}},\;M_{yx}=-2D_{33}\frac{\partial^{2}w}{\partial y\partial x},$$

where $D_{ij}$ is the flexural (bending) stiffness of the biological tissue, which is related to the elasticity moduli, Poisson’s ratio, and tissue thickness by

(14) $$\begin{array}{c} \langle D_{11},{\;D}_{22},{\;D}_{21},{\;D}_{12}\rangle =\frac{h^{3}}{12\left(1-\nu_{12}\nu_{21}\right)}\langle E_{11},{\;E}_{22},{\;\nu }_{12}E_{22},{\;\nu }_{12}E_{22}\rangle ,\\ D_{33}=\frac{G_{12}h^{3}}{12}, \end{array}$$

From the above equation, it is found that there is a direct proportional relationship between the bending stiffness and elasticity modulus. Using Hamilton’s principle, the couple resultants are related to the mass inertia and transverse displacement by

(15) $$\frac{\partial^{2}M_{yy}}{\partial y^{2}}+\frac{\partial^{2}M_{xx}}{\partial x^{2}}+2\frac{\partial^{2}M_{yx}}{\partial y\partial x}\;=m_{0}\frac{\partial^{2}w}{\partial t^{2}}-m_{2}\frac{\partial^{4}w}{\partial t^{2}\partial x^{2}}-m_{2}\frac{\partial^{4}w}{\partial t^{2}\partial y^{2}},$$

where the mass inertia coefficients *m*_0_ and *m*_2_ are given by

(16) $$m_{0}=\rho_{bt}h,m_{2}=\rho_{bt}\frac{h^{3}}{12}.$$

Inserting Equation (13) into Equation (15) yields

(17) $$-\left[D_{22}\frac{\partial^{4}w}{\partial y^{4}}\right.\left.+D_{11}\frac{\partial^{4}w}{\partial x^{4}}+2\left(2D_{33}+D_{12}\right)\left(\frac{\partial^{4}w}{\partial y^{2}\partial x^{2}}\right)\right]\;=m_{0}\frac{\partial^{2}w}{\partial t^{2}}-m_{2}\frac{\partial^{4}w}{\partial t^{2}\partial x^{2}}-m_{2}\frac{\partial^{4}w}{\partial t^{2}\partial y^{2}}.$$

## 4. Viscoelasticity Theory

Biological tissues contain a significant amount of fluid within individual cells, the extracellular matrix, and the interstitial regions, leading them to exhibit viscoelastic behavior [51,52]. Viscoelastic materials display both viscous and elastic properties when they are subject to mechanical loading. A number of different models of viscoelasticity including Kelvin–Voigt, Maxwell, and standard linear solid models have been used to analyse biological tissues [51]. The Kelvin–Voigt model uses a viscous damper parallel to an elastic spring, where the strain in the two elements are identical while the total stress is the sum of the stress in viscous and elastic elements. By contrast, in the Maxwell model, the elastic spring is connected to the viscous damper in series, leading to identical mechanical stress in each element. However, the total strain is the sum of strain in each element. In addition, the standard linear solid model has been used to describe the viscoelasticity in biological tissues [53]. In this model, a dashpot and two springs are used either in the form of a Maxwell representation or Kelvin representation to describe the time-dependent deformation behavior under applied loading. In this section, the Kelvin–Voigt model is used to derive the governing differential equations of biological tissue in a two-dimensional environment for the time-dependent transverse deformation.

Elasticity moduli given by *E_ij_* are required to be substituted by $E_{ij}\left[g\left(\frac{\partial }{\partial t}\right)(*)+(*)\right]$ in which g denotes the viscoelasticity constant of the biological tissue. As a result, bending stiffness components *D_ij_* are also needed to be substituted by $D_{ij}\left[g\left(\frac{\partial }{\partial t}\right)(*)+(*)\right]$ in Equation (13), leading to the following relations

(18) $$M_{xx}=-D_{11}\left(g\frac{\partial^{3}w}{\partial x^{2}\partial t}+\frac{\partial^{2}w}{\partial x^{2}}\right)-D_{12}\left(g\frac{\partial^{3}w}{\partial y^{2}\partial t}+\frac{\partial^{2}w}{\partial y^{2}}\right),\;M_{yy}=-D_{12}\left(g\frac{\partial^{3}w}{\partial x^{2}\partial t}+\frac{\partial^{2}w}{\partial x^{2}}\right)-D_{22}\left(g\frac{\partial^{3}w}{\partial y^{2}\partial t}+\frac{\partial^{2}w}{\partial y^{2}}\right),\;M_{xy}=-2D_{33}\left(g\frac{\partial^{3}w}{\partial x\partial y\partial t}+\frac{\partial^{2}w}{\partial x\partial y}\right).$$

Let us assume that the biological tissue is embedded by a viscoelastic medium with shearing, Winkler, and viscoelasticity effects. The force exerted by the viscoelastic medium onto the unit area of the biological tissue is

(19) $$f_{ve}=-c_{f}\frac{\partial w}{\partial t}+k_{s}\frac{\partial^{2}w}{\partial y^{2}}+k_{s}\frac{\partial^{2}w}{\partial x^{2}}-k_{w}w,$$

where *c_f_*, *k_s_*, and *k_w_* are, respectively, the viscoelasticity, shearing, and Winkler constants of the medium. By inserting Equation (18) into Equation (15) and utilising Equation (19), the final governing equation for the viscoelastic behaviour is obtained as

(20) $$-\left[D_{11}\left(g\frac{\partial^{5}w}{\partial x^{4}\partial t}+\frac{\partial^{4}w}{\partial x^{4}}\right)+D_{22}\left(g\frac{\partial^{5}w}{\partial y^{4}\partial t}+\frac{\partial^{4}w}{\partial y^{4}}\right)\right.\;\left.+2\left(D_{12}+2D_{33}\right)\left(g\frac{\partial^{5}w}{\partial x^{2}\partial y^{2}\partial t}+\frac{\partial^{4}w}{\partial x^{2}\partial y^{2}}\right)\right]\;-c_{f}\frac{\partial w}{\partial t}+k_{s}\frac{\partial^{2}w}{\partial y^{2}}+k_{s}\frac{\partial^{2}w}{\partial x^{2}}-k_{w}w\;=m_{0}\frac{\partial^{2}w}{\partial t^{2}}-m_{2}\frac{\partial^{4}w}{\partial t^{2}\partial y^{2}}-m_{2}\frac{\partial^{4}w}{\partial t^{2}\partial x^{2}}.$$

Equation (20) incorporates viscoelasticity effects on the time-dependent deformation of the biological tissue induced by both damping within the tissue structure and external damping due to the surrounding medium.

## 5. Poroelasticity Theory

In the poroelasticity theory, the biological tissue is modelled by assuming a material with a porous structure. The pores are assumed to be filled with fluid. When a mechanical load is applied to the structure, fluid flows within the pores and the biological tissue is deformed. Here, a mathematical model of poroelasticity is developed for biological tissue containing a spherical tumour. Using the mass conservation law for the fluid part, one obtains [54]

(21) $$\nabla \cdot \left(V_{f}n\rho_{f}\right)=-\frac{\partial }{\partial t}\left(n\rho_{f}\right),$$

in which *n* is the porosity, and $V_{f}$ is the fluid velocity. For the solid part, we have

(22) $$\nabla \cdot \left(\rho_{s}\left(1-n\right)V_{s}\right)=-\frac{\partial \rho_{s}}{\partial t}+\frac{\partial }{\partial t}\left(n\rho_{s}\right).$$

where index ‘*s*’ is employed to indicate the solid part of the biological tissue. Neglecting the compressibility of both fluid and solid parts and integrating the above mass conservation relations leads to

(23) $$\nabla \cdot V_{s}+\nabla \cdot \Phi_{d}=0,$$

In Equation (23), $\Phi_{d}$ represents the specific discharge, which is calculated by $\Phi_{d}=nV_{re}.$ Here, $V_{re}$ is the relative fluid velocity with respect to the solid part: $V_{re}=V_{f}-V_{s}.$ The solid matrix’s volume strain is

(24) $$\varepsilon =\varepsilon_{ii}={\left(u_{s}\right)}_{i,i}.$$

Here, ${\left(u_{s}\right)}_{i,i}$ is the first derivative of the displacement component ${\left(u_{s}\right)}_{i}$ with respect to the spatial coordinate *x_i_*. By employing Equations (23) and (24), one derives the following relation

(25) $$\nabla \cdot \Phi_{d}=-\frac{\partial \varepsilon }{\partial t}.$$

Darcy’s law states that there is a proportional relationship between the gradient of the fluid pressure within the pores of the tissue and the specific discharge as [54]

(26) $$\Phi_{d}=-\frac{{Κ}_{pm}}{\mu_{f}}\nabla p+\frac{{\rho_{f}Κ}_{pm}}{\mu_{f}}g,$$

where *p* stands for the fluid pressure and g is the gravity vector. Moreover, the fluid viscosity and tissue permeability are, respectively, denoted by $\mu_{f}$ and ${Κ}_{pm}$. By inserting the equation of Darcy’s law into Equation (25) and incorporating tissue microfiltration effects [55], the combined conservation mass equation is

(27) $$\frac{\partial \varepsilon }{\partial t}+\Upsilon_{tot}p=\frac{H_{c}}{\gamma_{vw}}\nabla^{2}p,$$

$\nabla^{2}$ denotes the Laplacian operator. The coefficients on the right-hand side of the above equation are given by $H_{c}={Κ}_{pm}\gamma_{vw}/\mu_{f}$ and $\gamma_{vw}=g\rho_{f}$. Also, $\Upsilon_{tot}$ represents the total microfiltration constant and is defined as $\Upsilon_{t}=\Upsilon_{va}+\Upsilon_{ly}$ [55,56]. Here, $\Upsilon_{va}$ and $\Upsilon_{ly}$ are the vascular (*va*) and lymphatic (*ly*) microfiltration constants of the solid lesion, respectively.

(28) $$\langle \Upsilon_{va},\Upsilon_{ly}\rangle =\langle \frac{k_{va}S_{va}}{V_{va}},\frac{k_{ly}S_{ly}}{V_{ly}}\rangle ,$$

*k* is the permeability while *V* and *S* are the volume and surface area of the corresponding part (i.e., *va* or *ly*). In the poroelasticity theory, the total normal stress ($\sigma_{ij}$) is the sum of the normal effective stress (${\sigma \prime }_{ij}$) and fluid pressure while the total shear stress is equal to the effective shear stress. This association for a spherical solid lesion can mathematically be expressed as

(29) $$\begin{array}{c} {\sigma \prime }_{rr}=\sigma_{rr}-p,\\ {\sigma \prime }_{\theta \theta }=\sigma_{\theta \theta }-p,\\ {\sigma \prime }_{\phi \phi }=\sigma_{\phi \phi }-p,\\ {\sigma \prime }_{r\theta }=\sigma_{r\theta },\\ {\sigma \prime }_{r\phi }=\sigma_{r\phi },\\ {\sigma \prime }_{\theta \phi }=\sigma_{\theta \phi }. \end{array}$$

The effective stress components are the parts of the total stress that directly cause the distortion and compression of the solid skeleton of the tissue. In the early detection of abnormal solid lesions within biological tissues, it is generally assumed that the mean radius of the spherical lesion is much lower than the side length of the tissue [56,57]. It is assumed that a spherical solid lesion is located within a biological tissue section. Distant loading is applied to the tissue section, causing the lesion to undergo mechanical compression. In ultrasound elastography imaging, this distant loading is induced by ultrasound transducers. As long as the lesion radius is small compared to the tissue dimensions, we can assume a uniform symmetric load along the radial direction on the solid lesion surface. According to this symmetric condition and by using the equilibrium equations of a finite element of the system together with Equation (29), one obtains

(30) $$\frac{2}{r}{\sigma^{\prime }}_{rr}-\frac{2}{r}{\sigma \prime }_{\theta \theta }+\frac{\partial {\sigma \prime }_{rr}}{\partial r}=-\frac{\partial p}{\partial r},$$

Here, *r* is the radial coordinate component. The effective stress components of the solid lesion can be written as

(31) $${\sigma \prime }_{rr}=-\left(2\mu \varepsilon_{rr}+\lambda \varepsilon \right),$$

(32) $${\sigma \prime }_{\theta \theta }=-\left(2\mu \varepsilon_{\theta \theta }+\lambda \varepsilon \right),$$

where μ is the first Lamé coefficient, also known as the shear modulus, while λ is the second Lamé coefficient. The strain components are

(33) $$\langle \varepsilon_{rr},\;\varepsilon_{\theta \theta },\varepsilon_{r\theta }\rangle =\langle \frac{\partial u_{r}}{\partial r},\frac{u_{r}}{r},0\rangle ,$$

(34) $$\varepsilon =\frac{\partial u_{r}}{\partial r}+\frac{2u_{r}}{r}=\frac{1}{r^{2}}\frac{\partial }{\partial r}\left(r^{2}u_{r}\right).$$

Here, $u_{r}$ is the biological lesion’s radial displacement. Using Equations (30)–(34), the final equilibrium equation is

(35) $$\left(\lambda +2\mu \right)\left(-\frac{2}{r^{2}}u_{r}+\frac{2}{r}\frac{\partial u_{r}}{\partial r}+\frac{\partial^{2}u_{r}}{\partial r^{2}}\right)=\frac{\partial p}{\partial r}.$$

In view of Equation (34), the combined mass conservation equation for the whole system including the solid and fluid parts is obtained as

(36) $$\frac{\partial }{\partial t}\left(\frac{2u_{r}}{r}+\frac{\partial u_{r}}{\partial r}\right)+\Upsilon_{tot}p=\frac{H_{c}}{\gamma_{vw}}\left(\frac{\partial^{2}p}{\partial r^{2}}+\frac{2}{r}\frac{\partial p}{\partial r}\right).$$

Equations (35) and (36) have been used in recent studies in order to improve the accuracy and resolution of ultrasound elastography imaging in detecting solid tumours [56].

## 6. Nonlocal Continuum Mechanics

Classical continuum mechanics is based on the fundamental assumption of stress locality. This assumption is valid at large-scale levels where the interaction between the individual microscopic components of the structure is negligible. By contrast, at the small-scale level, these interactions and the impact of the internal structural configuration become important [58,59]. Nonlocal continuum mechanics does not assume a local association between the stress components and the strain within the biological tissue. Instead, stress at a given point depends on the strain components at all domain points, allowing for the incorporation of stress nonlocality [60,61]. Moreover, an internal characteristics length is introduced by the nonlocal continuum theory, taking into account the effect of the internal structural configuration of the biological tissue at small-scale levels. In the context of biology and biomechanics, the small-scale level can be interpreted as the cellular level. Recent experimental measurements [62] and theoretical data [63] suggest that nonlocal continuum models may be well-suited to characterizing the biomechanical properties of biological tissue at small-scale levels.

At the cellular level, cancer cells exhibit a softer mechanical response while healthy normal cells display a more rigid response [62]. This softening behavior cannot be described using classical local continuum mechanics. However, the stress nonlocality assumption and the internal characteristics length are associated with a softer structural stiffness [64,65,66,67], enabling the description of cancer-associated softening behavior. Using Eringen’s nonlocal mechanics [68,69,70], the constitutive equation of the classical elasticity theory can be modified as

(37) $$\sigma_{nl}={∭}_{V}\psi_{nl}\left(\left|x\prime -x\right|,\chi_{mor-cell}\right)C:\varepsilon \left(x\prime \right)dV(x\prime ),$$

$\sigma_{nl}$, $\psi_{nl}$, and $\chi_{mor-cell}$ indicate the nonlocal mechanical stress, nonlocality modulus, and morphological–cellular parameters, respectively. The elastic matrix and strain parameter are denoted by C and $\varepsilon \left(x\prime \right)$, respectively [71,72]. The geometrical features of a clump of cells or region of biological tissue are captured by V, and $\left|x\prime -x\right|$, which are, respectively, the volume and distance between the two points x′ and x. As the volumetric integral in the right-hand side of Equation (1) makes it challenging to use this equation for further formulation and description of the biomechanical behaviour, an approximate differential form is introduced as [68,73,74]

(38) $$\left[1-{\left(e_{0}\mu_{mor}\eta_{cell}\right)}^{2}\nabla^{2}\right]\sigma_{nl}=C:\varepsilon ,$$

where $e_{0}$, $\mu_{mor}$, $\nabla^{2}$, and $\eta_{cell}$ represent the calibration parameter, morphological parameter, Laplace operator, and biomechanics cell parameter, respectively. $e_{0}$ is a constant to match the results of clinical experiments to those of mathematical modelling using the nonlocal continuum biomechanics. The small-scale morphological parameter is a cellular level feature, which describes how cells and extracellular components come together and form a larger biological structure. As an example, this parameter could be taken as the average distance between two adjacent cells. $\eta_{cell}$ is a combination of a number of biomechanical cellular features. The Laplace mathematical operator helps to incorporate cellular interactions and features as it takes higher derivatives of nonlocal stresses, and it is defined as

(39) $$\nabla^{2}f=f_{,jj},$$

where f is an arbitrary function and the subscript ‘,*_jj_*’ means taking the derivative of this function with respect to the independent variable ‘*j*’ twice. The morphological–cellular parameter is related to the calibration parameter, morphological constant, and biomechanical cellular features by

(40) $$\chi_{mor-cell}=\frac{e_{0}\mu_{mor}\eta_{cell}}{l_{ext}}.$$

Here, $l_{ext}$ is the external characteristic length. The definition of this length depends on the geometry of the biological sample. It can be taken as the length of the tissue section when the shape of the section is of rectangular one or $l_{ext}$ is the average radius of a spherical tumour when the biological samples are approximately spheres.

Using the nonlocal continuum mechanics and poroelasticity theory, the governing equation of spherical solid tumours under a uniform symmetric load along the radial direction can be obtained as [75]

(41) $$\begin{array}{c} -\frac{2\lambda }{r}\left(\frac{u_{r}}{r}\frac{\partial^{2}u_{r}}{\partial r^{2}}-\frac{\partial u_{r}}{\partial r}\right)+\left(2G+\lambda \right)\frac{\partial^{2}u_{r}}{\partial r^{2}}\\ +\frac{4G}{r}\frac{\partial u_{r}}{\partial r}-\frac{4G}{r^{2}}u_{r}-\frac{\partial p}{\partial r}+{\left(e_{0}\mu_{mor}\eta_{cell}\right)}^{2}\left(\frac{2}{r}\frac{\partial^{2}p}{\partial r^{2}}+\frac{\partial^{3}p}{\partial r^{3}}\right)\\ -\frac{1}{r^{2}}{\left(e_{0}\mu_{mor}\eta_{cell}\right)}^{2}2\left(\lambda +2G\right)r\left(\frac{\partial^{3}u_{r}}{\partial r^{3}}+\frac{4}{r}\frac{\partial^{2}u_{r}}{\partial r^{2}}\right)\\ -\left(\lambda +2G\right){\left(e_{0}\mu_{mor}\eta_{cell}\right)}^{2}\left(-\frac{4}{r^{2}}\frac{\partial^{2}u_{r}}{\partial r^{2}}+\frac{4}{r}\frac{\partial^{3}u_{r}}{\partial r^{3}}+\frac{\partial^{4}u_{r}}{\partial r^{4}}\right)\\ +\frac{2}{r^{2}}{\left(e_{0}\mu_{mor}\eta_{cell}\right)}^{2}\left\{\frac{\partial p}{\partial r}-{\left(e_{0}\mu_{mor}\eta_{cell}\right)}^{2}\left(\frac{2}{r}\frac{\partial^{2}p}{\partial r^{2}}+\frac{\partial^{3}p}{\partial r^{3}}\right)\right\}\\ +\frac{4}{r}{\left(e_{0}\mu_{mor}\eta_{cell}\right)}^{2}\left\{\frac{\partial^{2}p}{\partial r^{2}}-{\left(e_{0}\mu_{mor}\eta_{cell}\right)}^{2}\left(-\frac{2}{r^{2}}\frac{\partial^{2}p}{\partial r^{2}}+\frac{2}{r}\frac{\partial^{3}p}{\partial r^{3}}+\frac{\partial^{4}p}{\partial r^{4}}\right)\right\}\\ +{\left(e_{0}\mu_{mor}\eta_{cell}\right)}^{2}\left\{\frac{\partial^{3}p}{\partial r^{3}}-{\left(e_{0}\mu_{mor}\eta_{cell}\right)}^{2}\left(\frac{4}{r^{3}}\frac{\partial^{2}p}{\partial r^{2}}-\frac{4}{r^{2}}\frac{\partial^{3}p}{\partial r^{3}}+\frac{2}{r}\frac{\partial^{4}p}{\partial r^{4}}+\frac{\partial^{5}p}{\partial r^{5}}\right)\right\}\\ -\frac{2}{r}{\left(\lambda +2G\right)\left(e_{0}\mu_{mor}\eta_{cell}\right)}^{2}\left(\frac{\partial^{3}u_{r}}{\partial r^{3}}+\frac{4}{r}\frac{\partial^{2}u_{r}}{\partial r^{2}}\right)\\ +\frac{4}{r^{2}}{\left(e_{0}\mu_{mor}\eta_{cell}\right)}^{2}\left\{\frac{\partial p}{\partial r}-{\left(e_{0}\mu_{mor}\eta_{cell}\right)}^{2}\left(\frac{\partial^{3}p}{\partial r^{3}}+\frac{2}{r}\frac{\partial^{2}p}{\partial r^{2}}\right)\right\}\\ +\frac{2}{r}{\left(e_{0}\mu_{mor}\eta_{cell}\right)}^{2}\left\{\frac{\partial^{2}p}{\partial r^{2}}-{\left(e_{0}\mu_{mor}\eta_{cell}\right)}^{2}\left(-\frac{2}{r^{2}}\frac{\partial^{2}p}{\partial r^{2}}+\frac{2}{r}\frac{\partial^{3}p}{\partial r^{3}}+\frac{\partial^{4}p}{\partial r^{4}}\right)\right\}\\ +\frac{2}{r^{2}}{\left(e_{0}\mu_{mor}\eta_{cell}\right)}^{2}\left\{-\frac{\partial p}{\partial r}+4G\frac{\partial^{2}u_{r}}{\partial r^{2}}+{\left(e_{0}\mu_{mor}\eta_{cell}\right)}^{2}\left(\frac{2}{r}\frac{\partial^{2}p}{\partial r^{2}}+\frac{\partial^{3}p}{\partial r^{3}}\right)\right\}=0. \end{array}$$

Here, *G* denotes the shear modulus of the tumour and λ is the second Lamé coefficient. Equation (41), together with the conservation mass equation (36), can be used to obtain the distribution of displacement within the tumour. It can be concluded that stress nonlocality significantly changes the equilibrium equation of solid tumours by adding extra terms of radial displacement and pressure whereas the conservation mass equation of solid tumours is not affected. The governing equations of time-dependent deformation of solid tumours at large scales are obtained from Equation (41) by setting the nonlocal terms equal to zero (i.e., $e_{0}\mu_{mor}\eta_{cell}=0$).

## 7. Surface Acoustic Waves: Rayleigh and Scholte Waves

In addition to shear and longitudinal waves, other kinds of acoustic waves can be propagated within biological tissue. In this section, a brief overview is given on common types of surface acoustic waves including the Rayleigh wave and Scholte wave. Surface acoustic waves are widely generated and utilised in ultrasound elastography imaging in order to extract the mechanical characteristics of superficial tissues [76]. Various surface acoustic waves can be generated when the biological tissue is subject to different loading and boundary conditions. The general wave equation can be written as

(42) $$(\lambda +G)\nabla \nabla \cdot u+G\nabla ²u=\rho \ddot{u}$$

where λ and G are Lamé coefficients; u is the vector of displacements; ρ represents mass density; and $\ddot{u}$ denotes the second partial derivative of displacement vector with respect to time. Additionally, ∇² and ∇ are the Laplacian and gradient operators, respectively. The above equation is obtained based on four assumptions: (1) isotropic material, meaning that mechanical properties are the same along different directions, (2) homogeneous material, indicating that the mechanical features are consistent within different points of the target area, (3) linear relationship between stress and strain, and (4) there is no body force. According to the Helmholtz decomposition law [77,78], we have

(43) u=∇φ+∇×F,

where φ and F are a scaler potential and a vector potential, respectively. The vector potential is required to satisfy the following relations:

(44) ∇·F=0,

(45) ∇·∇×F=0.

Using Equations (42)–(45), one can obtain the following equations:

(46) $$\nabla^{2}\varphi =\frac{1}{c_{L}^{2}}\ddot{\varphi },$$

(47) $$\nabla^{2}F=\frac{1}{c_{T}^{2}}\ddot{F},$$

where

(48) $$\frac{1}{c_{L}^{2}}=\frac{\rho }{\lambda +2G},$$

(49) $$\frac{1}{c_{T}^{2}}=\frac{\rho }{G},$$

in which $c_{L}\;and\;c_{T}$ are, respectively, the longitudinal and transverse wave speeds. Rayleigh waves are generated and monitored at the tissue–air interfaces and are utilised to assess the mechanical properties of superficial tissues [79,80]. These waves are particularly helpful in the detection of abnormalities and disease conditions in lung [81], eye [82], and skin [83] tissues where a sufficient amount of the tissue–air interface is available to produce Rayleigh waves. In this type of wave propagation, particles of the tissue move in an elliptical path [76]. This elliptical motion is more pronounced at the top surface of the tissue and gradually decreases as the depth increases. The amplitudes of the oscillatory elliptical motion of particles decay in an exponential manner with the tissue depth [76,84].

Let us consider a linear isotropic tissue whose top surface is in contact with air. It is assumed that the surface acoustic wave is generated at the air–tissue interface along the *x* direction. In this case, the scaler potential and vector potential are obtained as

(50) $$\varphi =\varphi \left(x,z,t\right),$$

(51) $$F=F_{y}e_{y},$$

where $e_{y}$ is the standard unit vector along the *y* direction. It is worth mentioning that in the above relations, *z* is the coordinate component that is perpendicular to the tissue surface. Using Equations (50) and (51) together with Equations (43)–(47), the displacement components are obtained as

(52) $$u=\left(\frac{\partial \varphi }{\partial x}-\frac{\partial F_{y}}{\partial z}\right)e_{x}+\left(\frac{\partial \varphi }{\partial z}+\frac{\partial F_{y}}{\partial x}\right)e_{z}.$$

The amplitudes of the oscillatory motion of tissue particles decay in an exponential way with depth [76,84]. Thus, the non-zero components of the scaler potential and vector potential can be expressed as

(53) $$\varphi \left(x,z,t\right)=Aexp(-pz)exp[i\left(kx-\omega t\right)],$$

(54) $$F_{y}=Bexp(-sz)exp[i\left(kx-\omega t\right)].$$

Here, A and B denote the amplitudes of Rayleigh waves. The wave decay coefficients are denoted by *p* and *s*. Additionally, k and ω represent the wave number and frequency, respectively. Using Equations (53) and (54) together with the free boundary condition at the top surface, one can obtain the dimensionless displacement components of Rayleigh waves as follows

(55) $$\bar{u}=\frac{2k}{s^{2}-k^{2}}\left(-\frac{s^{2}+k^{2}}{2p}exp\left(-pz\right)+sexp\left(-sz\right)\right)e_{x}\;+\frac{2k}{s^{2}-k^{2}}\left(\frac{s^{2}+k^{2}}{2k}exp\left(-pz\right)-kexp\left(-sz\right)\right)e_{z}$$

Here, $\bar{u}$ is the dimensionless displacement vector that has been normalised by the amplitude of the *z*-component of the displacement at the top surface.

In addition to Rayleigh waves, Scholte waves are employed in biomedical imaging using surface acoustic wave elastography. This type of acoustic waves is generated at the tissue–liquid interfaces and provides a detailed mechanical characterisation analysis of the tissue of interest. Both superficial and deep tissue mechanics assessments can be conducted using Scholte waves [85]. As deeper tissue assessment is achievable in this technique [85], it has been successfully used for the diagnosis of liver diseases [76,86]. Let us take into account Scholte waves that are propagated along a tissue–liquid interface with linear isotropic material properties. Positive values of the *z* coordinate components indicate the solid region while the negative values of *z* represent the liquid region. The ultrasound Scholte waves are also induced and propagated within the liquid part, which can be formulated using the following relation:

(56) $$\varphi_{l}\left(x,z,t\right)=Cexp(-p_{l}z)exp[i\left(kx-\omega t\right)],$$

where C, $\varphi_{l}$, and $p_{l}$ are the amplitude of waves, scaler potential, and the wave decay coefficient of the liquid part, respectively. The above equation is only valid within the liquid region (i.e., *z* < 0) while Equations (53) and (54) are applicable in the solid region (i.e., *z* ≥ 0). Using Equation (56) together with the wave governing equations, one obtains the displacement of the tissue under Scholte waves as

(57) $$\bar{u}=\frac{k}{p\left(s^{2}-k^{2}\right)}\left(\left(s^{2}+k^{2}\right)exp\left(-pz\right)-2psexp\left(-sz\right)\right)e_{x}\;+\frac{1}{s^{2}-k^{2}}\left(\left(s^{2}+k^{2}\right)exp\left(-pz\right)-2k^{2}exp\left(-sz\right)\right)e_{z}$$

For more details about the derivation process of the above equations as well as more information about ultrasonic surface acoustic wave elastography, particularly Rayleigh and Scholte waves, an interested reader is referred to the recent review paper on this type of ultrasound elastography by Masud and Liu [76].

## 8. Recent Advancements

Table 3 summarises the three commonly used continuum models including classical elasticity, viscoelasticity, and poroelasticity theories as well as two recently developed promising models of nonlocal continuum mechanics for ultrasound elastography applications. The classical model of elasticity includes the estimation of two intrinsic mechanical features—Young’s modulus and Poisson’s ratio. The independent mechanical parameters that appear in the governing equations are the displacement components (*u_i_*) and spatial coordinate components (*x_i_*). The classical continuum mechanics of local elasticity model is limited as it does not allow for the incorporation of microfiltration, fluid, and scale effects. However, its benefits include simplicity, ease of implementation, and relatively low computational costs. In the viscoelasticity theory, the effect of the fluid content of biological tissue is taken into consideration through the introduction of a new biomechanical parameter known as the viscoelastic constant (*g*). In addition, time-dependent terms are added to the governing equations (refer to Equation (20)). The limitation of viscoelasticity models is the lack of microfiltration and the inability to describe the mechanical deformation at small-scale levels (i.e., scale effects). In the poroelasticity theory, fluid effects are modelled by two mechanical parameters—fluid viscosity ($\mu_{f})$ and tissue permeability (${Κ}_{pm}$). Furthermore, both vascular and lymphatic microfiltration effects can be incorporated into the modelling ($\Upsilon^{*}$). In this theory of continuum mechanics, the governing equations are described in terms of the displacement components (*u_i_*) and fluid pressure (*p*), which both depend on time (*t*) and spatial coordinates (*x_i_*). The classical version of the poroelasticity theory is restricted in terms of nonlinearity and scale-dependency.

Nonlocal continuum mechanics-based models have been developed recently for elastography applications to take into account mechanical phenomena associated with deformation at the cellular level. Stress nonlocality and strain gradient effects are among the most common scale effects that influence the deformation response of materials at nanoscale and microscale levels. A cell-sensitive scale parameter and a morphological parameter are introduced in the nonlocal elasticity model, enabling the incorporation of stiffness softening at the cellular level. Recent experimental observation confirms that cancer cells are softer than normal healthy cells [87], which classical elasticity theories are not able to describe. By contrast, stress nonlocality is incorporated in the nonlocal elasticity model, which is associated with stiffness softening at small-scale levels [88,89]. However, the nonlocal elasticity is restricted as it lacks the influences of microfiltration and fluid effects, which can be incorporated into the nonlocal poroelasticity model. Moreover, the introduction of new parameters comes with inevitable challenges regarding experimental measurements, computational speed, and clinical implementation. Further research is required to pave the way for the application of nonlocal continuum-based models in elastography imaging.

There needs to be a reasonable balance between computational cost and the level of accuracy that is required for a specific clinical application. In circumstances where the speed of the elastography image construction is critical, such as in a surgical imaging application, the classical elasticity, viscoelastic, and simple poroelastic models are appropriate choices as they require less computational time and resources. However, the accuracy of the imaging can be improved with more complex mathematical models, and these may be suitable when there are fewer time constraints. For example, for imaging aimed to detect early malignancy where there is less time constraint, models with higher precision but more computational cost such as scale-dependent nonlocal elasticity and nonlinear hyperelastic models might be promising options.

Table 4 lists a number of different continuum models that have been developed and utilised for ultrasound elastography imaging. The evaluation metric, metric value, study model, and potential clinical application as well as the level of computational complexity of the models are also presented. Overall, the classical elasticity theory of local mechanics is the simplest model with fewer biomechanical features while the nonlocal viscoelasticity models are of the highest computational complexity. Higher levels of computational complexity require more biomechanical parameters to be experimentally determined, leading to more challenges and technical difficulties in the clinical implementation of the model. Sadigh et al. [90] conducted a quantitative ultrasound elastography analysis on the classical local elasticity and found that the specificity of this medical imaging tool was between 78% and 88% for breast cancer detection. Zhou and Zhang [51] investigated different viscoelastic models of elastography imaging using tissue-mimicking phantoms and concluded that the Fractional Voigt model outperformed other models of viscoelasticity with a residual error of around 1.05. In another study, Islam et al. [19] developed a poroelastic model and tested its accuracy using an orthotopic mouse model. The accuracy of the poroelastic model was about 99% in the detection of malignant lesions of a spherical shape while it dropped to 90% in lesions of different geometrical shapes. More recently, a nonlocal viscoelastic model has been developed and integrated with machine learning for elastography imaging applications [91]. In silico simulations have shown that this model has a very low mean square error in the detection of ovarian diseases [91]. However, further experimental studies are required to enable the clinical application of this advanced continuum model.

Advancements in continuum modelling have been incorporated into ultrasound elastography for a number of clinical applications (Table 5). In 1993, Cespedes et al. [92] used a classical elasticity model to analyse the results of ultrasound elastography obtained by a linear array transducer. They verified the model and experimental setup on muscle and breast tissue in vivo. In pure strain elastography where only strain components are visualised, continuum models are not required. Geometry models can be utilised in these scenarios [93]. However, a combination of geometry and continuum models has been used in many applications, even in strain elastography imaging [94], allowing for the simultaneous estimation of strain components and mechanical properties [95]. The classical elasticity, viscoelastic [96], and poroelastic [95] models were developed for elastography imaging in the late 1990s and early 2000s. Early experimental testing and the validation of viscoelasticity and poroelasticity were conducted on tissue-mimicking phantoms [97,98] while later studies investigated the feasibility of the extraction of viscoelastic and poroelastic features of skeletal muscle [99], blood clots [100], and the liver [52].

In addition to the calibration of continuum models for elastography imaging of different organs, attempts have been made to adjust the elasticity and viscoelasticity models for different imaging modalities such as ultrasound or magnetic resonance imaging [14], dual mode ultrasound elastography [101], shear wave [51], and quasi-static elastography [62]. Bied and Gennisson [102] developed a nonlinear elasticity model for ultrasound shear wave elastography and experimentally calibrated the model on tissue-mimicking phantom samples and ex vivo bovine and porcine muscular tissues. They showed that the nonlinear elastic constant has the potential to be utilised as a new biomarker for comprehensive muscle biomechanical characterisation. In another interesting study, Aichele and Catheline [103] demonstrated that taking the effects of fluid viscosity and porosity in the continuum modelling into account enhanced the estimation of the elasticity modulus of soft porous materials in shear wave elastography imaging. In 2021, Islam and his coworkers [104] developed a poroelasticity model in conjunction with an experimental approach to non-invasively estimate the temporal and spatial distribution of fluid flow parameters within tissue-mimicking phantom samples and in a mouse model for breast cancer diagnosis.

More recently, Kishimoto et al. [105] studied the extraction of viscoelastic properties of tissue-mimicking phantoms using six different elastography imaging techniques including transient, point, and 2D shear wave ultrasound elastography as well as magnetic resonance elastography imaging. They found that the bias and variability of results of shear wave elastography were associated with not only the imaging system but also with the probe used and depth of measurement. Moreover, the mean speed of the shear waves propagated within the viscoelastic phantom samples estimated by ultrasound shear wave elastography systems was about 20% greater than that of magnetic resonance elastography at the frequency of 60 Hz, which is a widely used frequency in clinical applications. Khan and Righetti [106] developed an accurate mathematical framework to estimate vascular permeability in solid tumours based on a poroelasticity continuum model for ultrasound elastography imaging.

Nonlinear continuum models [111] have been recently developed for ultrasound elastography imaging [107], mainly based on the hyperelasticity theory [112]. When the tissue or organ undergoes large deformation, it is recommended to use hyperelasticity rather than poroelasticity. Large deformation is referred to a situation where the displacement components are in the same order of magnitude as the tissue dimensions. By contrast, the poroelasticity theory assumes a linear relationship between the strain and stress components and is an appropriate choice when the deformation induced by ultrasound waves is small. When a hyperelastic model is applied, nonlinear hyperelastic constants are required to be estimated in addition to the linear constant [110]. This would generally increase both the computational costs and experimental expenses of the elastography imaging. However, nonlinear hyperelastic constants contain extra information about the mechanical behaviour of the tissue, which can provide useful clinical data [124]. The choice between a nonlinear or a linear continuum model depends on different factors including specific application, desired level of precision, and time constraints as well as experimental and computational resources. Linear models such as the poroelasticity, viscoelastic, and elastic models without geometrical nonlinearity are relatively simple and fast, requiring less computational and experimental resources. However, hyperelastic models are mathematically more sophisticated, leading to more time-consuming computations but more accurate results.

In addition to the classical elasticity [120], viscoelasticity [118], and poroelasticity [123] theories, the biphasic theory has been developed to describe the mechanical behaviour of biological tissue under external forces [114]. In this theory, it is assumed that the biological tissue consists of two distinct phases—a solid phase and a fluid phase. The interaction between these two phases is of particular interest in the biphasic theory. Compared to poroelasticity, which has a broader range of applications from the mechanics of soils and rocks to biomechanics, the biphasic theory is specific to the biomechanics of biological tissue [115]. This theory has the capability to describe the impacts of nonuniform blood perfusion and heterogeneous vasculature on solid lesions’ response to mechanical loading [115].

Another continuum model is Eshelby’s theory, in which the stress distribution is estimated within an elastic medium containing a small ellipsoidal inclusion. Eshelby’s theory has a wide range of applications, and recently, it has been successfully used in ultrasound elastography imaging [122]. The limitations of Eshelby’s theory include linearity assumption, geometrical shape of inclusion, and infinite surrounding medium assumption. The accuracy of this model reduces as the inclusion shape deviates from a perfect ellipsoid or large deformation occurs (i.e., violation of linearity assumption). Another fundamental assumption that might not be held true in some clinical applications is that the background healthy tissue is assumed to be very large compared to the lesion. However, for the early detection of solid tumours where the lesion is small compared to the surrounding tissue, Eshelby’s theory is a reasonable continuum modelling approach [104].

Recent experiments have demonstrated that cancer is linked to stiffness softening at the cellular level [87]. This finding is paradoxical to the fundamental concept of elastography imaging that stiffness hardening is associated with tumour development at the tissue-scale level. This paradox highlights the need for the development of new mathematical platforms that enable the incorporation of stiffness softening at small-scale levels. Recently, a higher-order nonlocal continuum model has been proposed for elastography imaging of breasts by Farajpour and Ingman [108]. New mechanical features known as cell-sensitive scale parameters have been introduced, enabling the description of stiffness softening at the cellular level. The nonlocal elasticity model has been developed based on the basic assumption that biomechanical stresses at a specific point are not only a function of the strain at that particular point but also a function of strain at all points. This nonlocality assumption allows for the modelling of the scale effects associated with stiffness softening. However, the nonlocal governing equation of the biological tissue involves more differential terms, increasing the computational cost of elastography imaging. Moreover, clinical calibration of nonlocal continuum models using human tissue samples would be more challenging than classical elasticity since accurate cellular-level measurements are required to estimate scale parameters [91].

Table 6 provides a summary of the current technologies used to extract the mechanical properties of biological tissues at small-scale levels. The size range of the experimental technique as well as its benefits and drawbacks are listed. The ability of ultrasound elastography [49,125] and magnetic resonance elastography (MRE) [126] is limited to the tissue-scale level. The μElastography is capable of estimating abnormalities as small as several micrometers on an elasticity map [127], depending on the application of an accurate mathematical platform. When in-plane forces are involved, microscale tweezers can provide highly accurate measurements of mechanical features [128,129]. Optical coherence elastography [130] is another viable solution for microscopic-level measurements required in nonlocal elastography. However, the ability of this technique is restricted by relatively small penetration depths and the lack of capability to distinguish between tissue elasticity and density. In addition, innovative technology has been recently developed to estimate the mechanical features of biological tissues at the cellular level during microenvironment evolution over time based on the use of thermo-responsive microgel probes [131]. However, this method is restricted due to scalability challenges and the limitation of the estimations to the local regions. For ultrasmall estimation of the mechanical properties at the nanoscale/microscale levels, scanning force microscopy [132,133] and microrheology [134,135] can be utilised. Microrheology is precise at the estimation of viscoelastic properties while scanning force microscopy gives a two-dimensional surface image of the mechanical properties including elastic constants. It is concluded that the choice of imaging technique used to estimate the mechanical properties of biological tissue depends on the scale. This is also the case for the continuum models. At large-scale levels (i.e., tissue scale), the classical continuum models such as poroelasticity, hyperelasticity, and viscoelasticity are valid while at the small-scale levels (microscopic and nanoscale), discrete models, and nonlocal continuum mechanics are generally recommended.

## 9. Future Directions

Although a number of advanced continuum models have been recently developed for ultrasound elastography imaging, further research is needed to calibrate these models for clinical applications. A challenge is that there are different thresholds of mechanical properties that define healthy and diseased tissues. For example, the elasticity modulus of a healthy normal breast (both fibroglandular and fat tissue) is about 3.2 kPa while for ductal carcinoma in situ (DCIS) it is 16.4 kPa [15]. By contrast, the mean threshold stiffness for malignancy of thyroid nodules obtained by shear wave elastography is reported as 85 kPa [136]. Further research, particularly clinical studies, is required to to optimise these models and obtain cut-off thresholds for different diseases and tissues

More research is required to develop scale-dependent continuum models in order to understand the mechanisms of mechanical deformation at the cellular level. The current nonlocal elasticity models that incorporate scale effects are restricted in terms of linearity assumption and differential form of stress nonlocality. Linear assumption is only valid when the ultrasound-induced deformations are small compared to the tissue sizes. In the differential form of stress nonlocality given by Equation (38), the mechanical stress is related to the strain components through a partial differential equation. By contrast, the integral form of nonlocal elasticity stress is linked to strain by an integral relationship like Equation (37). It has been recently demonstrated that nonlocal integral models are mathematically well-posed and can incorporate long-range scale effects [71,137]. More importantly, new experimental approaches are needed in order to precisely measure newly introduced scale parameters and cell features associated with mechanical properties at microscales. To the best of our knowledge, neither an experimental technique nor a clinical approach has been developed to determine the parameters of nonlocal elasticity theory for biomedical elastography imaging.

Another common challenge remains regarding the inverse calculation of the mechanical properties from the displacement data. The current approach is based on direct modelling in which the mechanical properties are known, and displacement components are required to be estimated. It is essential to build robust and rapid mathematical frameworks to inversely determine mechanical characteristics from experimentally measured displacements. This could facilitate the widespread clinical application of elasticity imaging by enhancing measurement accuracy and the reproducibility of data.

When developing a continuum model for elastography imaging, it is important to take into consideration the computational time of the model. In many clinical applications, rapid elastogram construction in real-time is the key to the success of the diagnostic system. Although some advanced continuum models have been developed in recent years with improved accuracy, there has been limited attention to their computational costs and resources. Further research and algorithm optimisation is required to reduce the computational time and make complex continuum models such as scale-dependent nonlocal elasticity and nonlinear hyperelastic models efficient for real-time clinical applications.

Finally, an important research gap is that the majority of the available continuum models including elasticity, viscoelasticity, and nonlocal theory assume that the region of interest in the target biological tissue is homogeneous. However, it is clear that diseased regions can be highly heterogeneous [138,139]. The development of continuum models incorporating the influence of tissue heterogeneity would pave the way for increased precision that could improve clinical applications of ultrasound elastography.

## 10. Conclusions

Classical elasticity theory provides a rapid, computationally effective, and easy-to-use mathematical model to estimate mechanical properties for clinical applications of ultrasound elastography. However, this model lacks the ability to incorporate fluid effects associated with interstitial and intracellular fluid or scale effects associated with cellular level biomechanics. In the viscoelasticity theory of continuum mechanics, fluid effects are simulated through a viscoelastic coefficient while poroelastic models describe fluid–solid matrix interactions within biological tissue and can be modified to incorporate vascular and lymphatic microfiltration effects. Nevertheless, more time-dependent mathematical terms and the presence of extra mechanical features make poroelastic models more computationally expensive and experimentally challenging. Nonlocal continuum mechanics-based models include scale effects, enabling the incorporation of various mechanisms of deformation at the microscopic level. However, the introduction of scale parameters increases the computational cost and complexity of continuum modelling and imposes practical difficulties in calibrating the resultant models.

Advances in mathematical models have exciting potential to improve the accuracy of ultrasound elastography. Challenges remain in computational speed, intrinsic tissue heterogeneity, and the experimental measurements required for scale-dependent models. Increased accuracy of ultrasound elastography imaging technology will expand its clinical utility for early detection of disease and monitoring of treatment response.

## Figures and Tables

**Table 1:** Different mechanical loading conditions with their corresponding parameters [44].

Loading Condition	Stress	Strain	Mechanical Property	Hooke’s Law	Property Dependency	Strain Deformation
Axial	$\sigma_{xx}^{cl}$	$\varepsilon_{xx}$	E	$\sigma_{xx}^{cl}=E\varepsilon_{xx}$	E=2(1+v)G	$\varepsilon_{xx}=\frac{\Delta l}{l_{0}}$
Shear	$\tau_{xy}^{cl}$	$\gamma_{xy}$	G	$\tau_{xy}^{cl}=G\gamma_{xy}$	$G=\frac{E}{2(1+v)}$	$\gamma_{xy}=\frac{\Delta x}{l_{0}}=tan(\Delta \theta )$
Hydrostatic	−p	$\varepsilon_{v}$	K	$p=K\varepsilon_{v}$	$K=\frac{E}{3(1-2v)}$	$\varepsilon_{v}=\frac{-\Delta V}{V}$

**Table 2:** Longitudinal and shear waves within biological tissue [20,46,48].

Wave Propagation	Wave Speed	Incompressible Medium	Particle Oscilation	Approximate Speed in Soft Tissue (m/s)	Imaging Techniques
Longitudinal waves	$c_{L}=\sqrt{\frac{K}{\rho }}$	K→∞	Parallel to the wave propagation direction	1540	1-D transient and B-mode
Shear waves	$c_{S}=\sqrt{\frac{G}{\rho }}$	G=E/3	Perpendicular to the wave propagation direction	1 to 10	Point and 2D shear wave ultrasound elastography

**Table 3:** A comparison study between different continuum models used in ultrasound elastography imaging.

Model	Mechanical Properties	Independent Parameters	Microfiltration	Fluid Effects	Scale Effects	Computational Time
Classical elasticity	E,ν	$u_{i},x_{i}$	No	No	No	Very Low
Viscoelasticity	E,ν,g	$u_{i},x_{i},t$	No	Yes	No	Low
Poroelasticity	$E,\nu ,\mu_{f},{Κ}_{pm},\Upsilon^{*}$	$u_{i},p,x_{i},t$	Can be incorporated	Yes	No	Medium
Nonlocal elasticity	E,ν, $\mu_{mor},\eta_{cell}$	$u_{i},x_{i}$	No	No	Yes	High
Nonlocal poroelasticity	$E,\nu ,\mu_{mor},\eta_{cell},\mu_{f},{Κ}_{pm},\Upsilon^{*}$	$u_{i},p,x_{i},t$	Can be incorporated	Yes	Yes	Very high

**Table 4:** Evaluation metric, study model, computational complexity, and potential application of various continuum models of elastography imaging.

Continuum Model	Evaluation Metric	Metric Value	Study Model	Computational Complexity Level	Potential Clinical Application
Classical local elasticity [90]	Specificity	78–88%	Human breast tissue	Simple	Solid tumours
Viscoelasticity [51]	Residual error	1.0529	Tissue-mimicking phantom	Intermediate	Soft biological tissues
Poroelasticity [19]	Accuracy	90%	Orthotopic mouse model	Intermediate	Solid tumours
Nonlocal viscoelasticity [91]	Test mean square error	4.3 × 10^−6^	In silico study	Complex	Ovarian diseases

**Table 5:** A summary of developments in continuum modelling of biological tissue for ultrasound elastography imaging applications. Recent advances are given more focus.

Authors	Year	Model	Ultrasound Elastography	Tissue
Cespedes et al. [92]	1993	Classical elasticity	Ultrasound elastography by linear array transducers	Muscle and breast in vivo
Korte et al. [93]	1998	A geometry model	Strain imaging	Human arteries
Konofagou et al. [95]	1999	Poroelasticity	Poroelastography	Tissue mimicking phantoms
Walker et al. [96]	2000	Viscoelasticity	Acoustic radiation force ultrasound elastography	Tissue mimicking phantoms
Insana et al. [94]	2004	Viscoelasticity	Strain imaging	Tumour microenvironment
Berry et al. [97,98]	2006	Poroelasticity	Strain imaging	Tofu as a suitable poroelastic material
Hoyt et al. [99]	2008	Viscoelasticity	Shear wave	Skeletal muscle
Schmitt et al. [100]	2011	Viscoelasticity	plane shear wave	Blood clot
Chen et al. [52]	2013	Viscoelasticity	Shear wave	Liver
Mousavi et al. [14]	2015	Classical elasticity	Ultrasound or magnetic resonance imaging	Tissue-mimicking phantom for prostate cancer
Hong et al. [101]	2016	Viscoelasticity	Dual mode	protein hydrogels
Zhou and Zhang [51]	2018	Viscoelasticity	Shear wave	Phantom
Goswami et al. [62]	2020	Nonlinear elasticity	Quasi-static and shear wave	Gelatin phantoms
Bied and Gennisson [102]	2021	Nonlinear elasticity	Shear wave	Phantom and ex vivo bovine and porcine muscular tissues
Aichele and Catheline [103]	2021	Poroelasticity and viscoelasticity	Shear wave	Liver and phantom
Islam et al. [104]	2021	Poroelasticity	Poroelastography	Phantom and mice breast model
Kishimoto et al. [105]	2022	Viscoelasticity	Transient, point and 2D shear waves	Phantom
Khan and Righetti [106]	2022	Poroelasticity	Poroelastography	mice datasets with triple negative breast cancer
Zhang et al. [107]	2022	Hyperelasticity	High-frequency ultrasound elastography	Cornea and ciliary body
Farajpour and Ingman [108]	2023	Higher-order nonlocal elasticity	In-plane waves	Breast cancer
Tang et al. [109]	2023	Classical elasticity	Strain elastography	Spinal cord injury using an in-vivo rabbit model
Khan et al. [110]	2023	Hyperelasticity and viscoelasticity	Quasi-static and dynamic	Tissue mimic phantoms
Pagé et al. [111]	2023	Nonlinear elasticity	Shear wave	Gelatin-agar phantoms
Kheirkhah et al. [112]	2023	Hyperelasticity	Quasi-static	Tissue-mimicking phantom
Khan et al. [113]	2023	Poroelastic	Poroelastography	A mice model of triple-negative breast cancer
Majumder et al. [114]	2023	A bi-phasic poroelastic model	Poroelastography	Polyacrylamide samples and breast mouse model
Dwairy et al. [115]	2023	Biphasic theory	N/A	Solid tumour
Kheirkhah et al. [116]	2023	Inversion-based classical elasticity	Strain imaging	Locally breast cancer
Tecse et al. [117]	2023	Viscoelastic	Reverberant shear wave	Plantar soft tissue and gelatine phantom
Gotschi et al. [118]	2023	Viscoelastic	Shear wave	Tendon
Duroy et al. [119]	2023	Classical elasticity	Quasi-static ultrasound elastography	Phantoms and breast tissues
Elmeliegy and Guddati [120]	2023	Elasticity modelling	Shear wave	In silico simulation
Farajpour and Ingman [91]	2024	Nonlocal viscoelasticity	Scale-dependent elastography	Ovarian cancer, breast cancer, and ovarian fibrosis
Osika and Kijanka [121]	2024	Viscoelasticity	Shear wave	Phantom
Majumder et al. [122]	2024	Eshelby’s theory of continuum mechanics	Compression elastography	Phantoms and orthotopic mouse model of breast cancer
Cihan et al. [123]	2024	Poroelastic	Shear wave	Chicken breast
Gautam and Arora [124]	2024	Hyperelasticity	Strain elastography	Subcutaneous adipose tissue and Muscle thickness

**Table 6:** Biomedical imaging techniques to measure mechanical properties and displacement components at small-scale levels.

Imaging Device	Scale Range	Scale Range (m)	Benefits	Drawbacks	Available Studies
Magnetic resonance elastography	Tissue-scale level	10^−4^–10^−3^	Non-invasive, entire organ assessment, quantitative	Bulky, relatively expensive, lack of cellular resolution, limited availability	[126]
Microscale tweezers	Microscale	10^−5^	Ability to apply in-plane forces with high precision	Restrictions in strain extraction and scalability	[128,129]
Thermo-responsive microgel probes	Microscale	10^−5^–10^−4^	Tracking mechanical features during microenvironment evolution over time	Restricted to local regions, scalability limitation, challenging validation	[131]
Microrheology	Nanoscale and microscale	10^−9^–10^−6^	Accurate viscoelasticity measurements	Scale restrictions (only microscales and local regions), incompatible with larger scales	[134,135]
Scanning force microscopy	Nanoscale and microscale	10^−9^–10^−6^	detailed and precise elasticity maps at nanoscale level	Destructive tissue preparation, only 2D surface imaging	[132,133]
μElastography	Microscale	10^−7^–10^−3^	3D elasticity maps, multiplane details, Scalability	Depth limitations, reduced mechanical strain sensitivity	[127]
Ultrasound elastography	Tissue-scale level	10^−4^	Non-invasive, mobile, widespread availability, inexpensive, measurement flexibility	Reduced spatial resolution, not applicable at cellular level, signal attenuation due to fluid content	[49,125]
Optical coherence elastography	Microscale	10^−5^–10^−4^	Strong biocompatibility and enhanced mechanical sensitivity	Depth restriction, lack of capability to distinguish between elasticity and density	[130]
